# Multimodal ultrasound imaging in the diagnosis of primary vaginal malignant melanoma: A case report

**DOI:** 10.1097/MD.0000000000049636

**Published:** 2026-07-03

**Authors:** Hao-Cheng Qin, Min-Yan Wang, De-Quan Ye, Jiang Zhu

**Affiliations:** a Department of Ultrasound, Women’s Hospital, Zhejiang University School of Medicine, Hangzhou, Zhejiang, China; b Department of Ultrasound, The First Affiliated Hospital of Kangda College of Nanjing Medical University, Lianyungang, Jiangsu, China.

**Keywords:** CEUS, primary vaginal malignant melanoma, ultrasound

## Abstract

**Rationale:**

Primary vaginal malignant melanoma (PVMM) is an extremely rare and aggressive gynecological malignancy with a poor prognosis. Early and accurate diagnosis is challenging, and conventional imaging modalities have limitations. This case report highlights the application value of a multimodal ultrasonographic approach for the preoperative assessment of PVMM.

**Patient concerns:**

A 48-year-old postmenopausal female with a long-term history of systemic sclerosis presented with a two-month history of vaginal bleeding and discharge. She reported a recent increase in discharge volume.

**Diagnoses:**

Gynecological examination revealed a firm, ulcerated mass on the posterior vaginal wall. Transrectal high-frequency ultrasonography, combined with multimodal techniques including three-dimensional ultrasound, superb microvascular imaging, shear-wave elastography, and contrast-enhanced ultrasound, characterized a hypoechoic mass with rich peripheral vascularity, specific stiffness patterns, and a “fast-in and fast-out” enhancement pattern. These findings were highly suggestive of malignancy, which was subsequently confirmed by biopsy and immunohistochemistry.

**Interventions:**

This report focuses on the diagnostic intervention, namely the comprehensive ultrasonographic evaluation using the aforementioned multimodal techniques. The patient underwent a biopsy of the vaginal mass for pathological diagnosis.

**Outcomes:**

The integrated multimodal ultrasound examination provided detailed structural, vascular, and functional information about the lesion, leading to a strong suspicion of malignancy that was pathologically confirmed as PVMM.

**Lessons:**

Multimodal ultrasonography, incorporating high-resolution imaging, microvascular assessment, elastography, and contrast enhancement, offers significant value in the diagnostic evaluation of PVMM. It can provide high-resolution anatomical details and quantitative functional data, potentially improving preoperative assessment and diagnosis of this rare tumor.

## 1. Introduction

Primary vaginal malignant melanoma (PVMM) is an extremely rare gynecological malignant tumor with an incidence rate of approximately 0.46 per million.^[1,2]^ It predominantly affects postmenopausal women, presenting challenges in early diagnosis and a poor prognosis.^[3]^ Currently, preoperative diagnosis mainly relies on conventional imaging modalities such as MRI; however, these techniques have limitations in assessing tumor microvasculature and mechanical properties.^[4]^ Traditional ultrasound examination offers limited diagnostic value for this disease and often provides insufficient information. Therefore, exploring novel multimodal ultrasound techniques is of great significance for improving the preoperative diagnostic accuracy of this condition.

## 2. Case report

**Figure 1.**
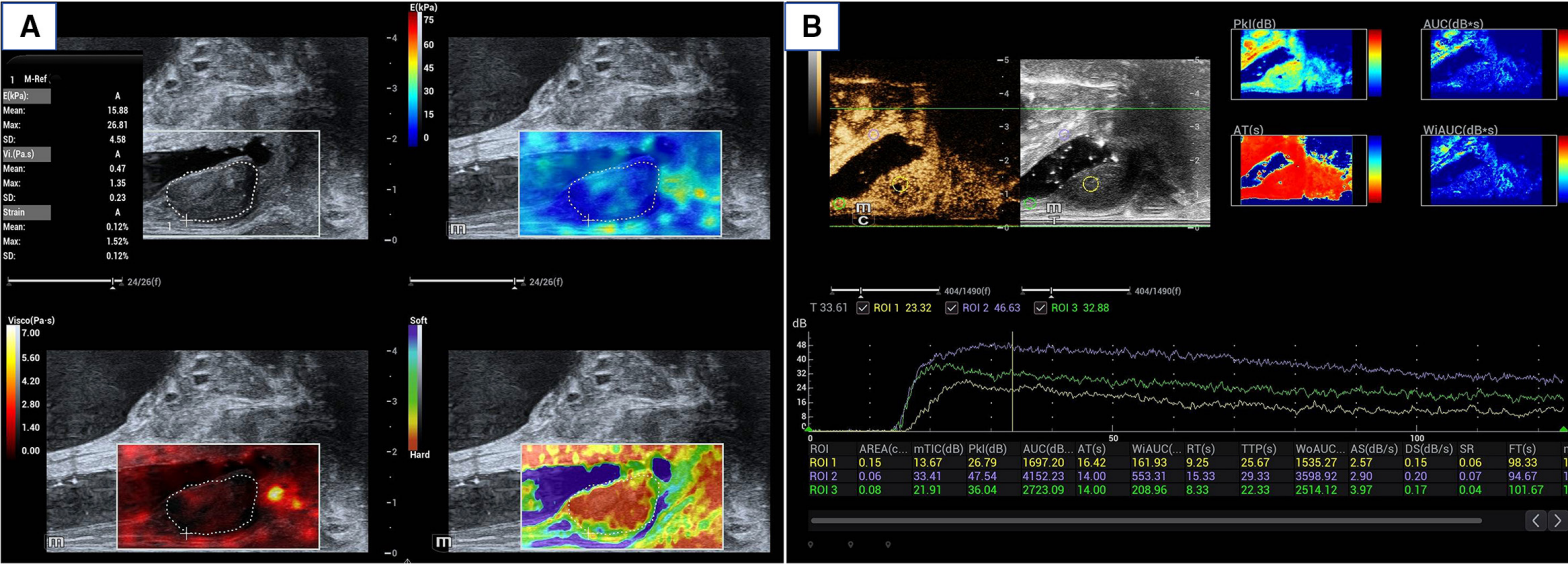
Multi-parameter combined imaging and CEUS images. (A) Shear wave elastography in the upper right, viscoelastic imaging in the lower left, and strain elastography in the lower right; (B) CEUS time-intensity curve (TIC). CEUS = contrast-enhanced ultrasound.

**Figure 2.**
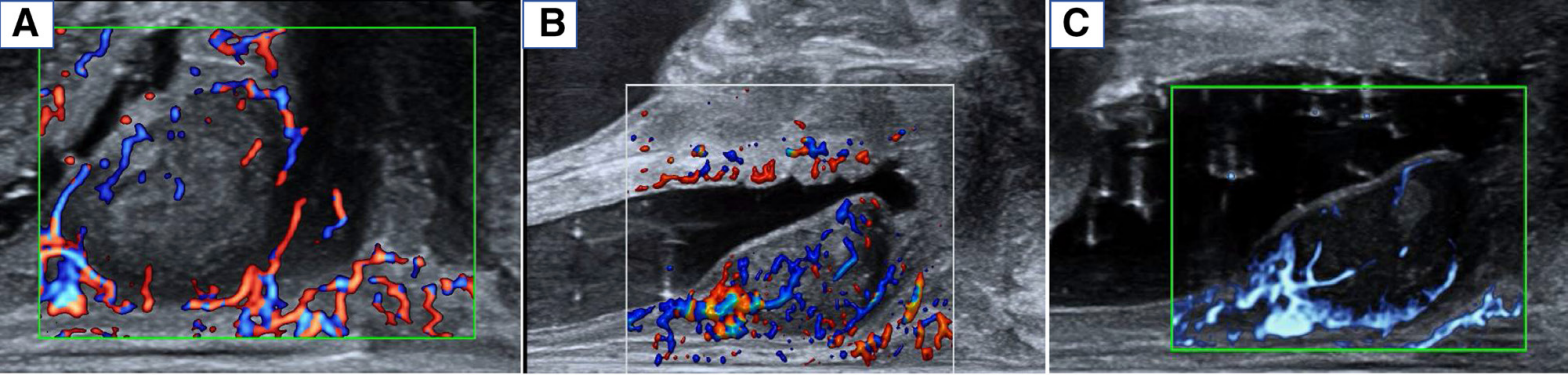
Superb microvascular imaging. (A) Power superb microvascular imaging; (B) Color superb microvascular imaging; (C) Subtraction superb microvascular imaging.

**Figure 3.**
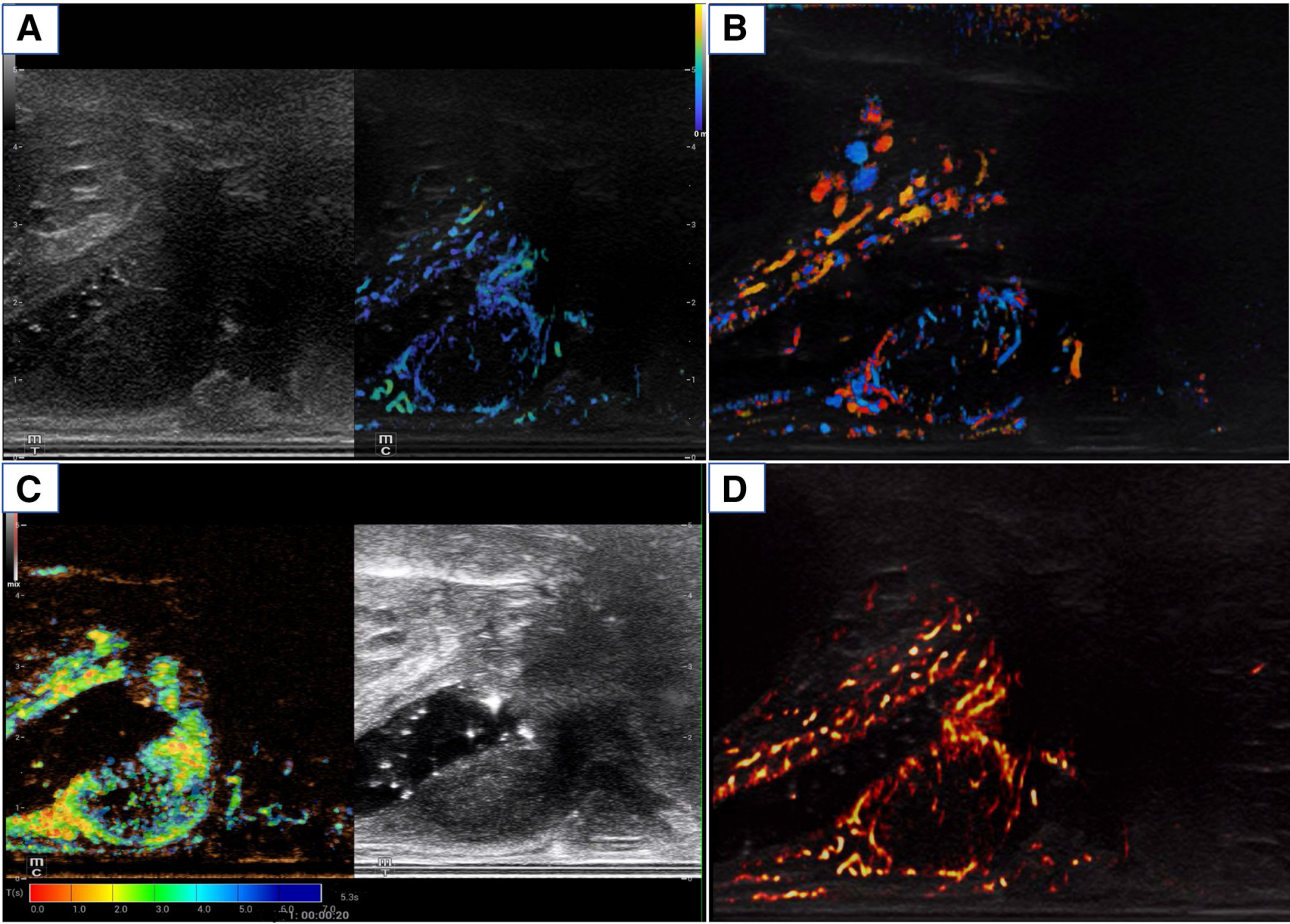
SR CEUS images. (A) Blood flow velocity map; (B) Blood flow direction map; (C) Temporal pattern map; (D) Vascular density map. SR CEUS = super resolution contrast-enhanced ultrasound.

**Figure 4.**
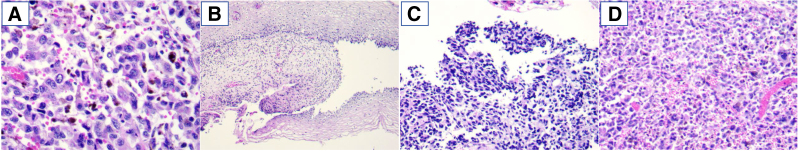
Pathological images.

A 48-year-old female was admitted to the hospital due to “postmenopausal vaginal bleeding and discharge for 2 months, and discovery of a vaginal mass for 1 day.” The patient had a 10-year-plus history of systemic sclerosis, for which she had received regular treatment and underwent periodic reexaminations. She reported comorbidities including pulmonary fibrosis, primary cirrhosis, and renal dysfunction. Two months ago, she developed a small amount of vaginal discharge without obvious inducement, which was clear with blood streaks and no obvious odor; the amount of discharge increased in the past week. Gynecological examination revealed a colorless mass with a diameter of 3 cm on the posterior and right lateral walls of the vagina, which was hard in texture, poor in mobility, with surface ulceration and bleeding on palpation. Transrectal high-frequency linear array ultrasound (using a 360° full-field volume probe) revealed a 2.2 × 1.8 cm × 1.2 cm hypoechoic mass on the posterior vaginal wall, with clear boundaries, regular shape, heterogeneous internal echotexture, and no obvious invasion of the rectum (Fig. S1A–C, Supplemental Digital Content 1). Using pelvic floor tomographic ultrasound imaging technology, the anatomical relationship between the mass and the urethra, rectum, and pelvic wall was clearly demonstrated on the sagittal, coronal, and transverse planes (Fig. S2A, B, Supplemental Digital Content 2). In the multi-parameter combined imaging mode: shear wave elastography Young modulus values were Max 26.81 kPa and Mean 15.88 kPa; viscoelastic imaging values were Max 1.35 Pa.s and Mean 0.47 Pa.s; strain elastography score was 4 points (Fig. 1A). Superb microvascular imaging (SMI) showed abundant circumferential blood flow around the mass with a resistive index of 0.74, which was confirmed by super resolution contrast-enhanced ultrasound (SR CEUS) vascular density map, vascular direction map, and temporal pattern (Figs. 2A–C and 3B–D). Contrast-enhanced ultrasound (CEUS) showed that the contrast agent began to fill the lesion on the posterior vaginal wall at the 16th second, reached the peak of enhancement at the 25th second, and exhibited a “fast-in and fast-out” enhancement pattern. The lesion presented hypoperfusion compared with the surrounding normal vaginal wall, and the relevant quantitative analysis results are shown in Figure 1B. SR CEUS indicated that the interior of the mass was dominated by low-velocity blood flow (Fig. 3A). Based on the above ultrasound features, a malignant tumor was considered possible. Both contrast-enhanced CT and MRI findings suggest that the mass is indicative of a malignant tumor (Fig. S3A–C, Supplemental Digital Content 3, Fig. S4A–C, Supplemental Digital Content 4). Complete set of tumor markers: human epididymis protein 4 was 82.60 pmol/L, premenopausal Rome Index was 20.66%, and postmenopausal Rome Index was 14.71%. The patient’s authorized representative fully understood the condition and requested only vaginal wall mass biopsy. Pathological diagnosis: malignant melanoma; immunohistochemistry: CgA(−), CK8/18(−), HMB45(+), SOX-10(+), Myogenin(−), CK7(−), LCA(−), PAX8(−), SMA(−), Ki-67(20%+), Melan-A(+), P53(70% positive, with varying intensities) (Fig. 4A–D).

## 3. Discussion

PVMM is an extremely rare type of cancer.^[1]^ It predominantly affects postmenopausal women, with clinical manifestations including vaginal bleeding, vaginal mass, and pain.^[5]^ The diagnosis of PVMM involves routine examinations such as clinical manifestations and palpation, and can also be supplemented with comprehensive analysis using ultrasound, CT, and MRI. In particular, CT and MRI enable more precise evaluation of pelvic wall status and lymph node metastasis. Characteristic MRI findings include isointense to hyperintense signals on T1WI, hypointense signals on T2WI, hyperintense signals on DWI, and obvious enhancement on contrast-enhanced scans. The clinical manifestations of this patient were typical, and the enhancement patterns on contrast-enhanced CT and MRI were consistent with previous reports.^[6]^

As the preferred screening method, ultrasound plays a crucial role in the early and accurate assessment of lesions through the flexible application of both traditional and novel technologies.^[7]^ However, there are few reports on ultrasound findings of this disease, and the limited available literature mainly focuses on the description of two-dimensional and color Doppler ultrasound features, which fail to provide sufficient ultrasonic imaging information for diagnosis. Therefore, we used multiple ultrasonic imaging techniques to evaluate this case, aiming to provide valuable insights for the ultrasonic diagnosis of PVMM.

For the ultrasonic imaging evaluation of vaginal lesions, both traditional transabdominal and transvaginal ultrasound have limitations: the former suffers from insufficient resolution, while the latter is restricted by scanning angles. However, the application of transvaginal high-frequency linear array probes can effectively overcome these limitations. Drawing on its successful application experience in superficial organs such as the thyroid and breast, this technique enables high-resolution visualization of the microanatomical layers of lesions through a posteroanterior scanning path, providing a novel perspective for clinical diagnosis.^[8]^ In this case, due to the small size and low location of the lesion, conventional transabdominal and transvaginal ultrasound failed to detect it effectively. In contrast, transrectal high-frequency linear array ultrasound not only clearly visualized the lesion itself but also offered distinct advantages in finely distinguishing the internal structure of the lesion and its relationship with surrounding tissues. Furthermore, three-dimensional images stereoscopically and intuitively demonstrated the anatomical border between the mass and the posterior rectum, providing crucial imaging support for clinical diagnosis and doctor-patient communication.

While MRI is indispensable for assessing the depth of tumor invasion, involvement of adjacent organs (such as the bladder and rectum), and pelvic lymph node status, it has limitations in evaluating the real-time microvascular architecture and hemodynamic characteristics of the tumor. The spatial resolution of MRI is limited, making it difficult to directly visualize microvascular structures, and it can only indirectly reflect perfusion and permeability. In contrast, ultrasound combined with advanced techniques offers several advantages. First, it enables real-time hemodynamic assessment using color Doppler and contrast-enhanced ultrasound. Second, ultrasound offers superior accessibility and cost-effectiveness, making it a valuable first-line screening tool for guiding biopsies and facilitating frequent follow-ups, particularly in resource-limited settings or for patients with contraindications to MRI. Therefore, ultrasound and MRI play complementary roles; ultrasound provides unique, real-time functional information about tumor vasculature that cannot be obtained through standard MRI sequences.

Detailed reports on the use of CEUS for evaluating PVMM are extremely scarce in the literature. Our application of SR CEUS to characterize the tumor’s microvascular architecture and hemodynamics represents a novel contribution to the imaging description of this rare disease. However, it should be noted that the “fast-in and fast-out” pattern observed on CEUS, while indicative of malignancy, is not a specific feature of melanoma. This pattern reflects tumor angiogenesis and arteriovenous shunting, which can be seen in various malignant tumors. Therefore, this finding must be considered as part of the diagnostic puzzle, and the final diagnosis should still rely on histopathology and immunohistochemical confirmation.

PVMM should be differentiated from other vaginal malignancies, including primary and secondary types. Primary vaginal malignancies are rare and include squamous cell carcinoma, adenocarcinoma, and sarcoma, among others. Secondary vaginal malignancies can result from direct extension of cervical cancer, with lesions primarily located in the cervix. The main symptoms include contact bleeding or irregular vaginal bleeding, while other symptoms are similar to those of squamous cell carcinoma, adenocarcinoma, and other malignancies.^[9]^ In this case, the high stiffness value strongly supports a malignant etiology, as benign lesions typically exhibit lower and more uniform elasticity. However, we acknowledge that elastography findings are not specific to melanoma and must be interpreted in conjunction with grayscale and Doppler features.

The clinical presentation, MRI, and CT features of this case are consistent with most reported cases of primary vaginal melanoma in the literature.^[10,11]^ The unique aspect of our case, however, lies in its comprehensive integration of multimodal imaging techniques, including shear-wave elastography, SMI, CEUS, and SR CEUS. A literature search confirms the current lack of detailed reports on the use of contrast-enhanced ultrasound in primary vaginal melanoma. This makes our case a valuable addition, demonstrating the potential of these advanced techniques in characterizing tumor stiffness and vascularity.

## 4. Conclusion

In summary, this case highlights the diagnostic challenges posed by PVMM. Our experience underscores the importance of a multimodal diagnostic approach. While histopathology combined with comprehensive immunohistochemistry remains the gold standard for diagnosis, advanced imaging techniques play an indispensable complementary role. This case demonstrates that integrating high-resolution imaging, microvascular assessment, elastography, and CEUS can provide quantifiable, objective reference points for the clinical diagnosis of PVMM, thereby mitigating the subjective limitations of traditional qualitative diagnosis.

## Acknowledgments

This work was financially supported by National Natural Science Foundation of China (grant numbers:82272004 to JZ), Lianyungang Health Technology Project (QN202203 to HCQ).

## Author contributions

**Conceptualization:** Hao-Cheng Qin, Jiang Zhu.

**Data curation:** Hao-Cheng Qin, De-Quan Ye, Jiang Zhu.

**Funding acquisition:** Jiang Zhu.

**Investigation:** Hao-Cheng Qin.

**Resources:** Jiang Zhu.

**Writing – original draft:** Hao-Cheng Qin.

**Writing – review & editing:** Min-Yan Wang, Jiang Zhu.








