# Supplementary figures and images for: Multimodal ultrasound imaging in the diagnosis of primary vaginal malignant melanoma: A case report
Source: Medicine (Baltimore). 2026 Jul 3;105(27):e49636. doi: 10.1097/MD.0000000000049636 (PMC13337010; doi:10.1097/MD.0000000000049636)

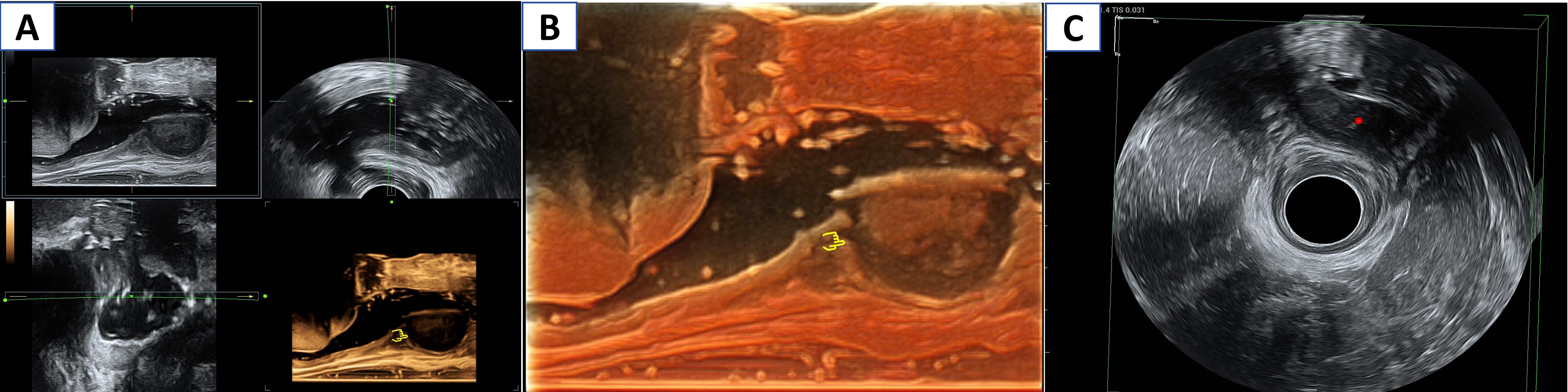

Supplement: Supplementary file 1 [file medi-105-e49636-s001.tif]

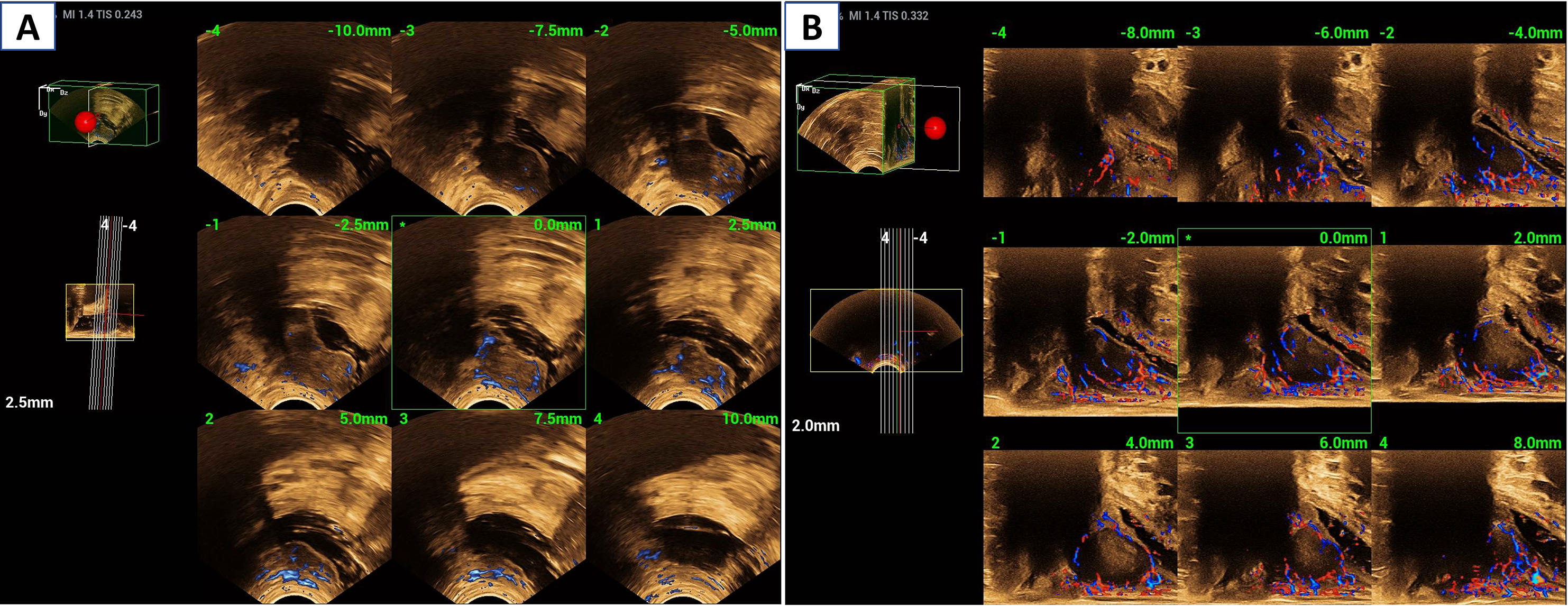

Supplement: Supplementary file 2 [file medi-105-e49636-s002.tif]

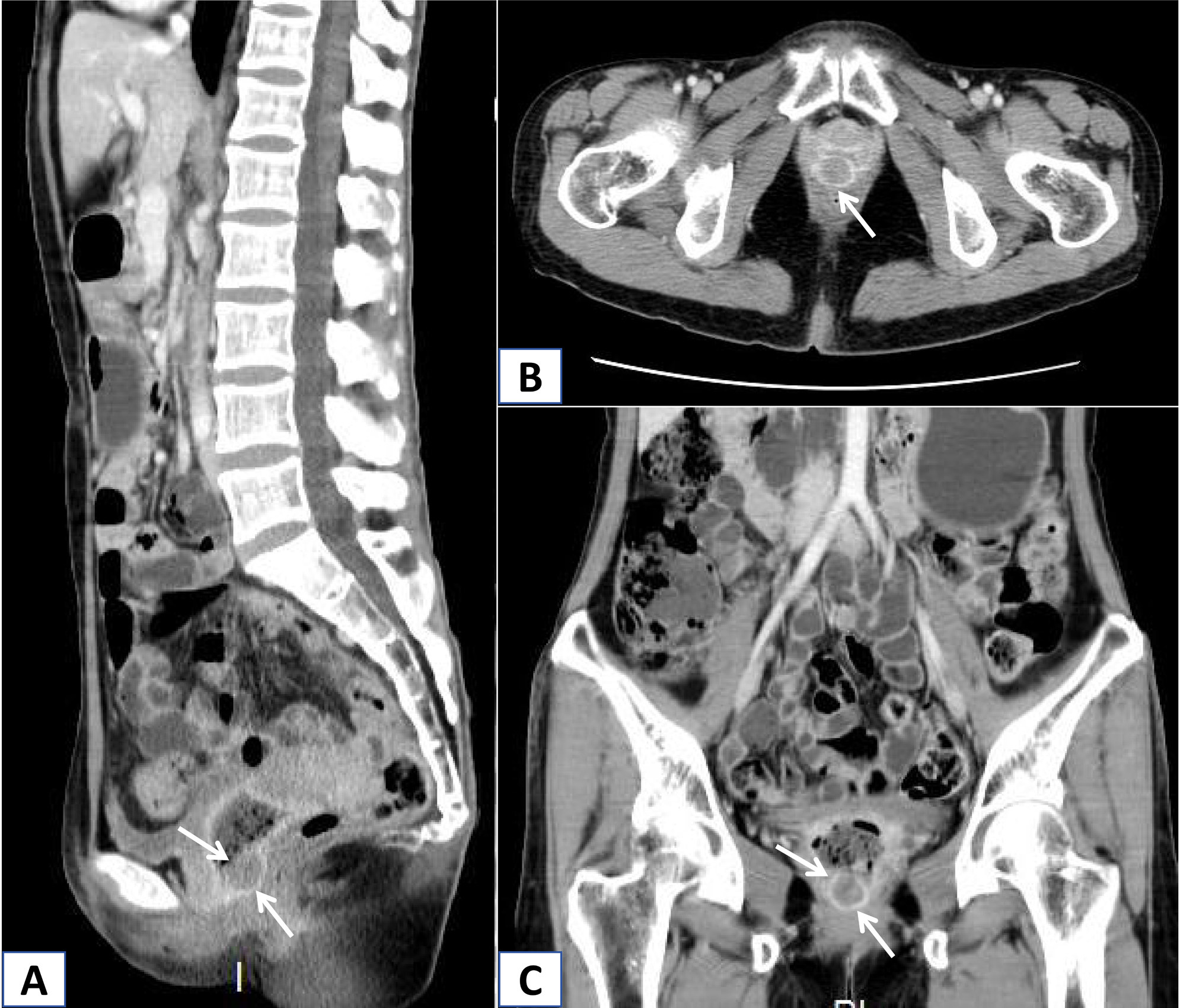

Supplement: Supplementary file 3 [file medi-105-e49636-s003.tif]

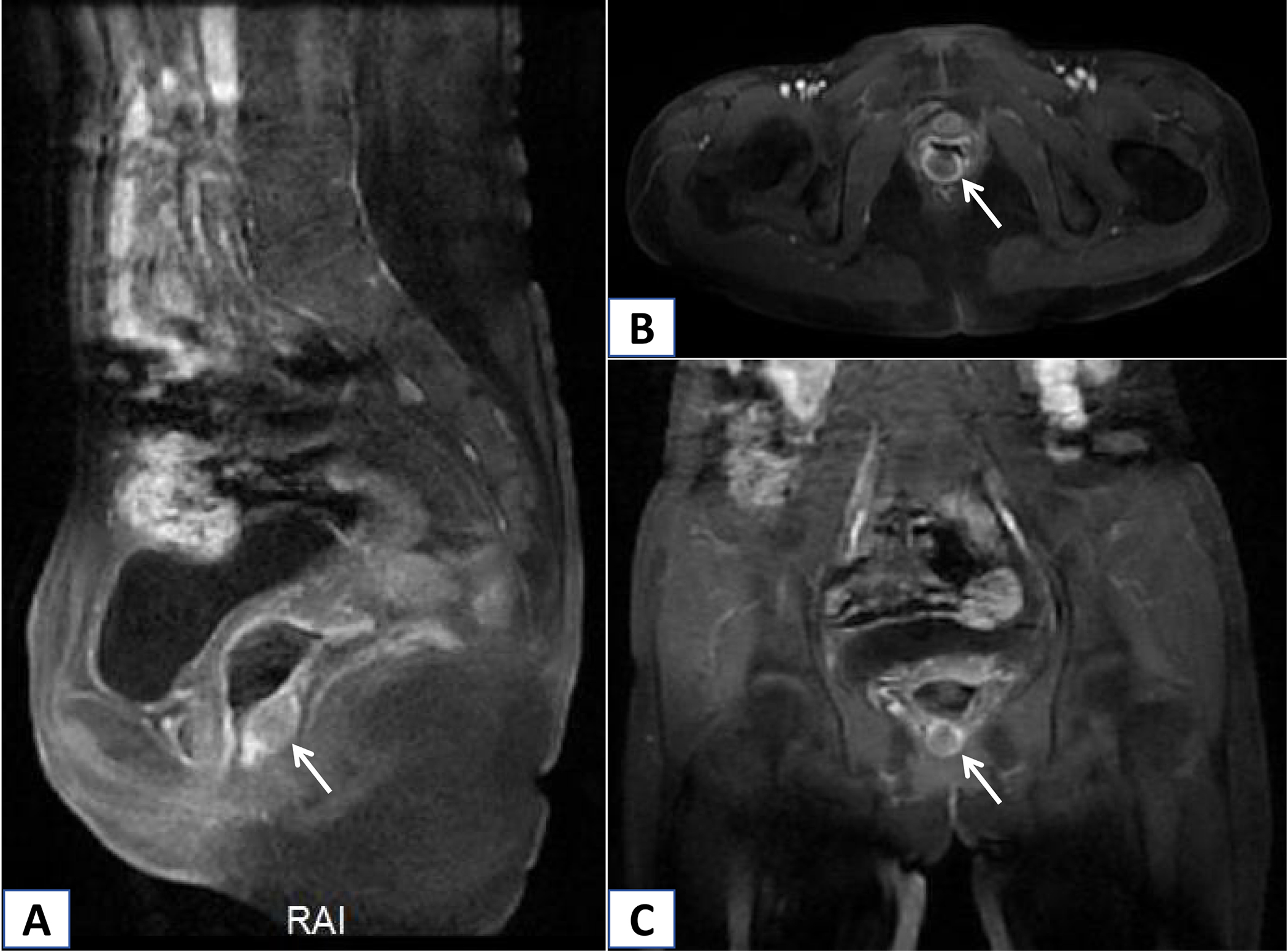

Supplement: Supplementary file 4 [file medi-105-e49636-s004.tif]
